# Health Monitoring System from Pyralux Copper-Clad Laminate Film and Random Forest Algorithm

**DOI:** 10.3390/mi14091726

**Published:** 2023-09-01

**Authors:** Chi Cuong Vu, Jooyong Kim, Thanh-Hai Nguyen

**Affiliations:** 1 Faculty of Electrical and Electronics Engineering, Ho Chi Minh City University of Technology and Education, 01 Vo Van Ngan Street, Linh Chieu Ward, Ho Chi Minh City 700000, Vietnam; cuongvc@hcmute.edu.vn; 2 Department of Materials Science and Engineering, Soongsil University, Seoul 156-743, Republic of Korea; jykim@ssu.ac.kr

**Keywords:** soft pressure sensor, water resistance, Pyralux copper-clad laminate, random forest, respiratory monitoring

## Abstract

Sensor technologies have been core features for various wearable electronic products for decades. Their functions are expected to continue to play an essential role in future generations of wearable products. For example, trends in industrial, military, and security applications include smartwatches used for monitoring medical indicators, hearing devices with integrated sensor options, and electronic skins. However, many studies have focused on a specific area of the system, such as manufacturing processes, data analysis, or actual testing. This has led to challenges regarding the reliability, accuracy, or connectivity of components in the same wearable system. There is an urgent need for studies that consider the whole system to maximize the efficiency of soft sensors. This study proposes a method to fabricate a resistive pressure sensor with high sensitivity, resilience, and good strain tolerance for recognizing human motion or body signals. Herein, the sensor electrodes are shaped on a thin Pyralux film. A layer of microfiber polyesters, coated with carbon nanotubes, is used as the bearing and pressure sensing layer. Our sensor shows superior capabilities in respiratory monitoring. More specifically, the sensor can work in high-humidity environments, even when immersed in water—this is always a big challenge for conventional sensors. In addition, the embedded random forest model, built for the application to recognize restoration signals with high accuracy (up to 92%), helps to provide a better overview when placing flexible sensors in a practical system.

## 1. Introduction

The sensor technology industry is expected to make great strides in the coming decades by adopting a wide range of wearable devices in everyday life [1,2]. Augmented and virtual reality devices [3,4,5], such as VR, AR, or MR, combine cameras, depth sensors, and tension or pressure sensors, allowing users to interact with specific content. Product categories such as smart clothing and other related products are all based on a core sensor, where communication between the human body and surrounding environments is a key function of the product. Various types of sensors are specific to different technologies and application contexts, in which the most significant proportion are chemical, optical, and electromechanical sensors [6,7,8,9,10,11].

Going deeper into the aspects and applications, we highlight some examples. Chemical sensors provide an alternative to needles [12]; they allow people with diabetes to monitor blood sugar without pricking a finger. Today, commercial devices are still mainly needles. Therefore, the quest to find new wearable sensors that are less invasive to the skin is ongoing. Motion sensors are used for pedometer applications [13,14]; most small wearables are set up with built-in accelerometers. Many motion-sensing components are included in clinical trials or track the movements of athletes to analyze and provide the most appropriate and quick solutions. Optical sensors are used in heart rate detection [15]. We are familiar with smartwatches with a light cluster on the back of the device, which is used to obtain heart rate data or blood oxygen, thereby enabling deeper analysis into calories consumed or sleep quality. Many companies are competing for the lead in commercializing blood pressure monitors [16]. Other companies are targeting devices in the form of ‘small clinics on the wrist’ that will replace conventional tests in hospitals [17]. The electrodes allow the monitoring of heart rate, muscles, and brain signals [18]. Incorporating conductive materials into wearable technology is a simple and established concept. However, this is the principle behind countless types of wearable sensors today, including soft, wet electrodes glued to the skin to measure heart rate, dry electrodes in headphones to analyze brain electrical signals, and microneedle tips in skin patches to quantify muscle movements. This also creates a wide range of applications for electrode sensors [19,20,21], from vital sign monitoring and sleep analysis for healthcare to emotional response or stress monitoring.

Among the flexible wearable sensors, the pressure sensor [22,23] has become an integral part of the tasks of detecting movements or muscle changes. The new generation of soft pressure sensors [24,25] exhibits distinct advantages such as being lightweight, small, hypoallergenic, comfortable to wear on the body, and suitable for many different locations and ranges. The above properties are achieved by the materials that make up the sensor, the most important of which are nanomaterials such as metal nanowires (NWs), carbon nanotubes (CNTs), conductive polymers, and nanoparticles (NPs). Flexible pressure sensors fabricated from CNTs and graphene oxide (GO) are attracting much attention from research groups worldwide [26,27]. Their applications appear in various research areas, including patient body signal recognition, athlete tracking, human–robot interface, and electronic textiles [28,29,30]. There has been a lot of research on prominent soft pressure sensors over the years. These studies have focused on one or a few aspects of flexible wearable sensors, such as materials or construction. The above studies lack evaluations when placing sensors in an overall wearable system. In addition, studies also lack efficient and intelligent signal processing models that can optimize sensor performance.

From the above approach, this study constructed a system to monitor human health signals through a specific application: respiratory detection masks. In this study, a flexible sensor was developed and considered when connected with other components of the wearable system, consisting of a processing board and electrode connections. On the other hand, an embedded machine-learning model was built to observe the data obtained from the system. The model helps to provide a better overview of when the flexible sensor is used in an actual healthcare application. In more detail, the study presents a pressure sensor that uses a Pyralux film for electrodes, and the sensing layer is wipe-coated with carbon nanotubes (CNTs). Here, the sensors are thin (0.26 mm) and highly sensitive at 0.2 kPa^−1^ (under 6 kPa)/0.05 kPa^−1^ (over 8 kPa). There are some highlights of this work. Firstly, the simple process helps the sensor retain the benefits of conventional resistive sensors and expands the limits of sensors in healthcare or electronic control devices (thin and high performance). Secondly, the sensor is water-resistant. This capability would suit many working environments and applications, such as body tracking systems, rain gear, and robots. Thirdly, the sensor structure can be hand-drawn without any design software intervention. More importantly, body signals are classified based on a combination of a multifunctional design, consisting of a new-generation flexible pressure sensor and an embedded machine-learning model built on the collected data. The sensors are expected to be applied in practice in clinics or hospitals. This is also a potential investment direction for companies or businesses in the near future.

## 2. Materials and Methods

Pyralux copper-clad laminate film was purchased from Dupont Ltd., Wilmington, DE, USA. The film consists of Kapton polyimide and is available in sheet form as a single-sided clad. The structure archives high flexibility and compatibility. The carbon nanotube (CNT) inks were 0.1 wt% solutions from KH Chemicals Company, Seoul City, Republic of Korea. The wet-etching material (sodium persulfate (Na_2_S_2_O_8_)) was obtained from SME Company, and the wet wipes were from SsangYong Company, Seoul, Republic of Korea. A spacer layer was constructed from a hot-melt adhesive film (PU film). The final sensor was protected and secured using a thin single-sided sticky film from 3M Technology Ltd., Seoul, Republic of Korea.

**Figure 1:**
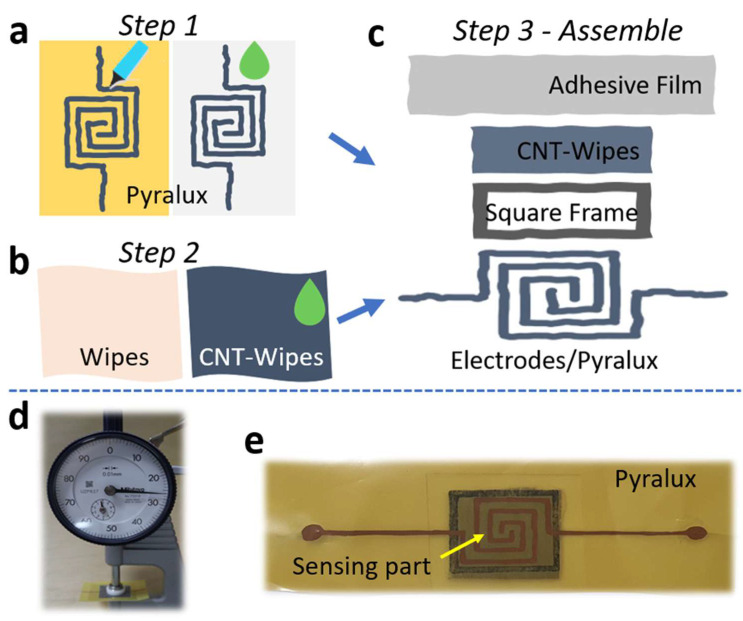
Manufacturing process of the flexible pressure sensors, consisting of (**a**) preparing the electrode layer, (**b**) preparing the sensing layer, (**c**) assembling sensor, (**d**) thickness of the sensor, and (**e**) real image of the final flexible sensor.

The manufacturing process of the flexible pressure sensors is shown in Figure 1a–c. There were three main steps: process the electrode layer, process the sensing layer, and assemble the final sensor. From a nonwoven master roll, wet wipes were fabricated via a converting process and contained multiple microfibers with a dimension of 10 µm arranged randomly together. They were dried at 100 °C for 10 min to remove the water, then dipped in conductive inks, squeezed, and dried again to obtain the dry wipe-coated CNTs. The CNT particles were attached to microfibers using the binder inside the inks and the interfiber force (friction). However, this adhesion is weak, and the CNTs could fall out when working in particle applications (large deformations). These CNT wipes had a sheet resistance of 27–30 Ω⁄sq and a thickness of 0.16 mm.

Pyralux film is a type of flexible printed circuit board (PCB) with a thickness of 0.032 mm. This film has two sides; one side is polyimide, and another is a copper layer. The electrodes were shaped with a permanent marker pen, and the distance between each electrode was about 1 mm. Then, the Pyralux film was dipped in etching liquid to remove nonelectrode parts for 25 min. After the wet-etching process, we used acetone liquid to clean the marker lines. Finally, the sensor was assembled from many parts. Figure 1c describes the structure, consisting of electrodes (Pyralux), a sensing layer (CNT wipes), a spacer film (double-sided hot melt adhesive film), and a cover layer (one-sided adhesive thin film). The total thickness was about 0.26 mm, and the size was 15 × 15 mm, as seen in Figure 1d,e. Obviously, the above structure has the potential to be applied in real-life cases.

## 3. Results and Discussion

**Figure 2:**
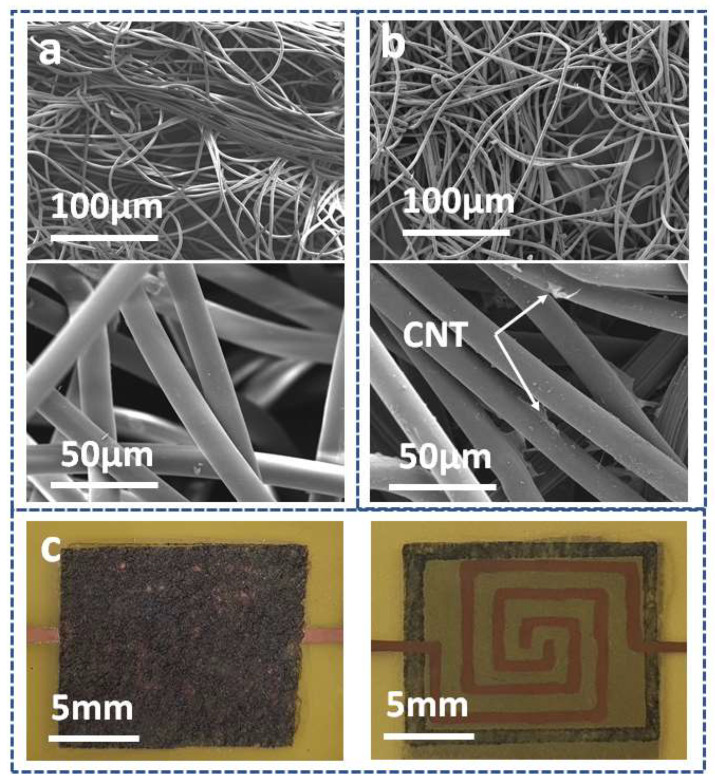
Scanning electron microscope images of the sensor: (**a**) sensing layer at 100 µm and 50 µm before dipping in CNTs, (**b**) sensing layer at 100 µm and 50 µm after dipping CNTs, and (**c**) assembled sensor at the top view and bottom view.

**Figure 3:**
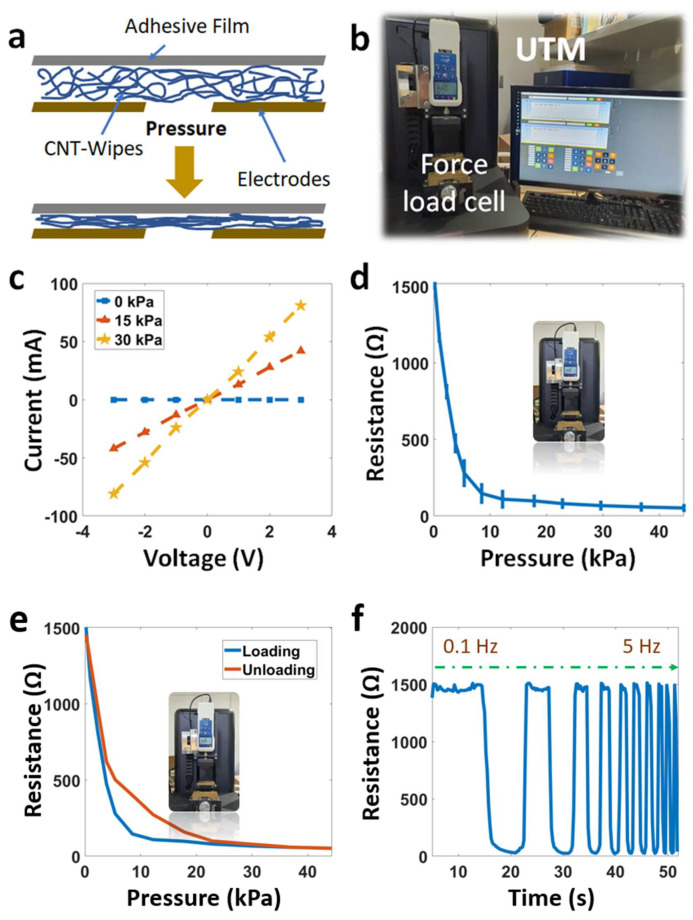
Characteristics of the sensor: (**a**) working principle, (**b**) universal testing machine, (**c**) current–voltage graphical curves, (**d**) resistance change under pressure, (**e**) hysteresis, and (**f**) resistance change at different frequencies.

Field-emission scanning electron microscope (FE-SEM) images of each layer are shown in Figure 2. The CNT wipes are a random structure of many fibers. There are many empty spaces between the fibers or bundle fibers, and each single fiber has a diameter of 10 µm. The conductive particles (CNTs) cover the single fiber or create some random film on the pristine surface of the wipes. Following the principle of the pressure sensor, our sensor converts the mechanical pressure value into an electrical resistance signal. As shown in Figure 3a, the thickness of the sensing layer (CNT wipes) decreases when applying force. The CNT arrays on the microfiber polyesters come closer together, increasing the conductivity of the sensing layer so that the overall resistance decreases. At that time, the conductivity between the electrode lines (copper) on the Pyralux layer increase. After removing the force, the CNT-wipe layer is released. The microfiber polyesters move further, causing the overall resistance to recover to its original value.

The working performances of the sensors were tested with a universal testing machine (UTM) (Figure 3b and Figure S1), which included a controlling computer, a force load cell (DN-FGA-20), and an LCR meter (Keysight E4980AL). The compression speed of the UTM system was 0.02 mm/s, and the resolution of the force gauge (FGA-K2) was 0.0098 N/cm^2^ with an error range of ±0.2%. All testing was performed at a room temperature of 25 °C. The sensor samples were located on the sole plate, and two electrodes were connected to two crocodile clips (soldering method) for reading the resistance signal. Figure 3c describes the current–voltage graphical curves (I–V) at different pressure levels from 0 to 50 kPa. These curves indicate an Ohmic behavior at voltages from −3 to 3 V. This parameter demonstrates good linearity for practical applications. Figure 3d,e show the resistance change in the sensor with increasing/decreasing force. In addition, the sensitivity is calculated as S = δ(△R/$R_{0}$)/δP, where $R_{0}$ is the initial resistance, R is the resistance under pressure, and the δP is the pressure change.

It is clear that the sensitivity was about 0.2 kPa^−1^ in the low-force range under 6 kPa and about 0.05 kPa^−1^ in the compression force range was over 8 kPa. This is due to the saturated contacting area at high pressure. Figure 3d also shows the sensor has a small standard deviation error (<7%). Hysteresis is defined as the deviation between the response/release resistance lines and dependence of the history states. Two hysteresis lines can be seen in Figure 3e, with a slight difference for pressure from 0 to 45 kPa. In Figure 3f, the sensor show excellent dynamic performance over a wide mechanical frequency (0.1–5 Hz).

**Figure 4:**
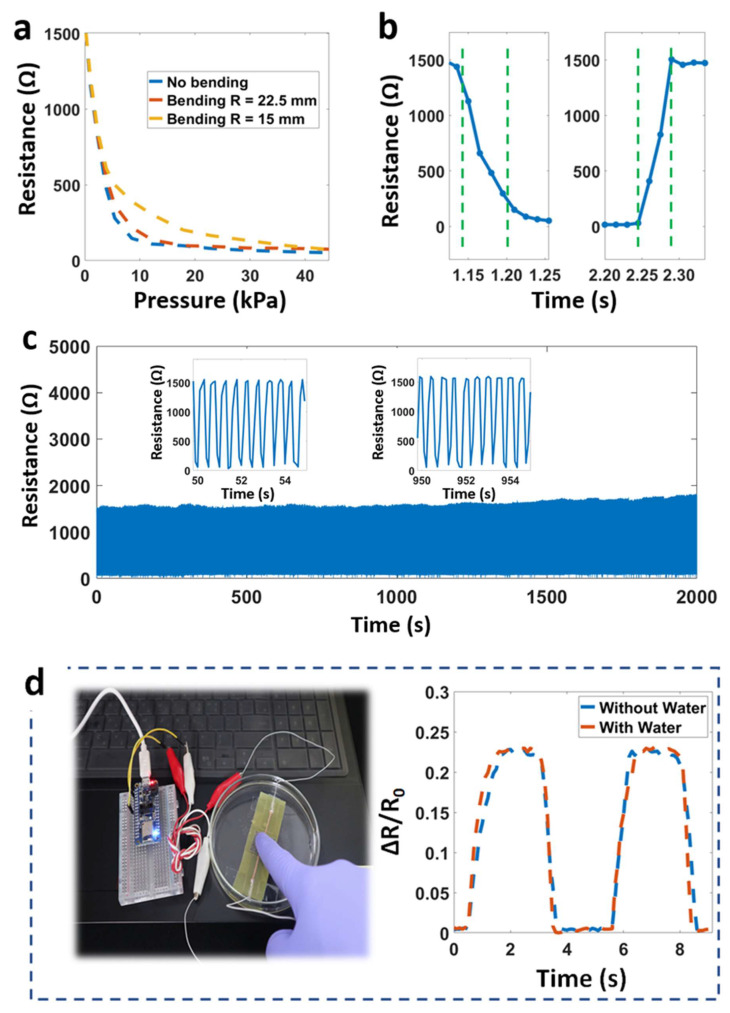
(**a**) Resistance change at different bending radii, (**b**) response and recovery times, (**c**) durability under loading/unloading cycles, and (**d**) working when dipped under water.

Figure 4 and Figures S2–S5 show essential characteristics, such as response/recovery time, resistance change at different frequencies, bending angles, temperatures. Due to the benefits of the chosen flexible materials, the sensor performed well in the working test at three different levels of radii (0–15–22.5 mm). Figure 4a describes the resistance change under bending deformations. Here, the workability diminishes with curvature change but is generally still suitable for the considered radii. The main reason for the delay is the connection time between CNT coating layers when applying pressure and the viscosity/elasticity of the fibers. We observed a short value at a response time of 70 ms and at a recovery time of 50 ms (Figure 4b). These above values ensure the electrical properties and the working potential of the sensor in monitoring and controlling cases.

Durability represents the ability of the sensor to remain functional when faced with many loading/unloading cycles. In this work, the sensor had stable responses and mechanical integrity under 3000 cycles. We obtained a small difference (maximum resistance) with 5% after 1000 cycles and about 8% after 2500 cycles. These changes were caused by the permanent deformation in the structure of the sensor when working, leading to a change in the maximum resistance. The CNT particles can fall out of the fibers, and cracks appear in the CNT-coated layers during operation. We recommend a solution for this issue: replacing the CNT inks with Ag or Au pastes. However, this approach (Ag/Au) will raise costs and change the sensor’s characteristics. Another sensor feature is the working capacity in high-humidity environments or directly underwater. As shown in Figure 4d, the sensor was immersed in water to a depth of about 1 cm (sensing part only). The result shows a slight change in the output signal. Figure S2 describes stable operation when immersed in water for different times (0, 6, or 12 h). The reason for this is that the sensing layer (CNT wipes) is entirely enclosed by two water-resistant layers, polyimide (from Pyralux), and 3M adhesive. Additionally, Table 1 summarizes some studies on flexible sensors, including principle, sensitivity, response/recovery time, and water resistance [22,31,32,33,34,35,36]. Our sensor clearly demonstrates good capabilities such as water resistance, short response time, and good sensitivity for a wide range of wearable applications.

## 4. Embedded Health-Monitoring System with Flexible Sensor and Random Forest Algorithm

**Figure 5:**
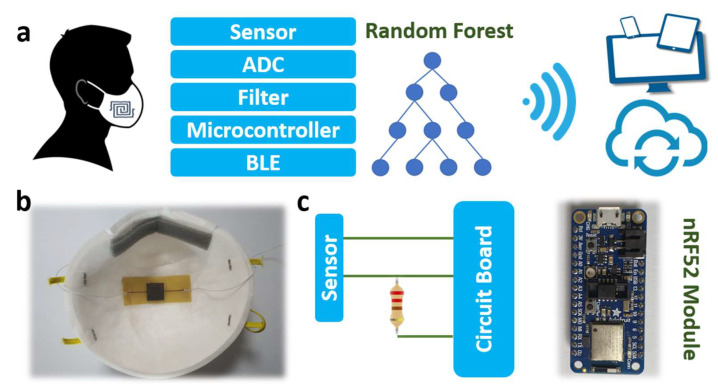
(**a**) Respiration checking with the embedded system, (**b**) smart mask with the flexible sensor, and (**c**) signal processing board with nRF52 module.

To demonstrate the ability of the fabricated sensor, we propose a method to monitor the breathing rate of humans (Figure 5). This indicator is significant for applications to analyze daily clinical health after exercise or for respiratory disease. The system consists of three main parts: a smart mask with a flexible pressure sensor, a processing circuit board, and a lipo-battery (3.7 V). An embedded circuit collects the signals with an nRF52 module, including an analog/digital converter (ADC), a microcontroller (MCU), and a Bluetooth low-energy (BLE) part. This module is a completely embedded board that can run a small AI model. Here, a random forest model was built to classify the types of respiration: normal breath, deep breath, rapid breath, cough, and breath holding. As described, we collected the signals via sampling, digitizing, and converting them into digital signals at a rate of 1000 signals per min from the system. One filter removes unusual vibrations and noises. Then, the features are extracted from the time- and frequency-domain breathing samples, including signal amplitude, standard deviation, breathing cycle, mean value, variance, root mean square, and unbiased estimation. These features are used for the random forest model [37,38]. Finally, the classified respiration data are sent via Bluetooth to phones, tablets, or smartwatches.

Random forest (RF) is a type of machine-learning algorithm based on decision trees and is commonly used for regression or classification problems. RF builds many decision trees, and each tree is unique. The final classifications are from the aggregation of these decision trees. This algorithm has proven particularly powerful for tasks with data from soft sensors. The application shows an overview of how the flexible sensor can be placed in a complete system containing many components. Here, we evaluated two main problems with flexible sensors when used in a system without considering the manufacturing process. Firstly, there is a connection between the sensors and the electrical wires. Soft sensors are not created from heat-resistant materials, so soldering the metal wires directly onto the electrode layers is not reasonable. The best practice is to secure part of the wire with strong adhesive and fix the rest of these wire ends with a laminating layer. Secondly, a suitable AI model is necessary for manipulating the obtained data. Wearables have an issue in that the average deep- or machine-learning models are too large to be applicable on a small embedded board. Therefore, choosing a suitable model for each application is extremely necessary. This leads to a concept—tiny machine learning for wearable devices. Basically, it takes a lot of experiments and tweaking of the system and signal acquisition method on the embedded board (programming) to obtain the best combination.

**Figure 6:**
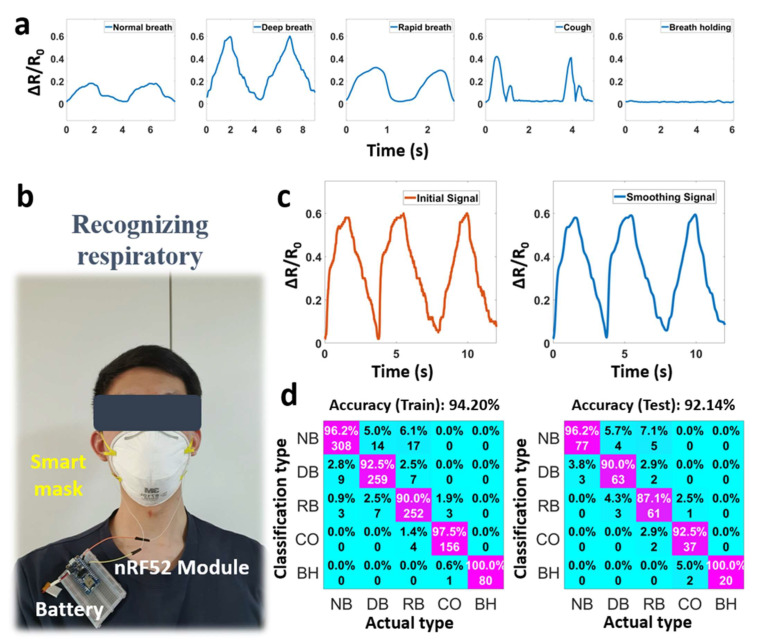
Respiratory monitoring system, consisting of (**a**) different breathing signals, (**b**) smart mask for recognizing respiratory, (**c**) initial and smoothing signal, and (**d**) confusion matrices.

Figure 6a,b show different breathing signals and a complete system, including a smart mask, flexible pressure sensor, and circuit board. Figure 6c describes the initial signal and smoothing signal. This paper considers some filters, such as Kalman, Butterworth, and average filters, to reduce the noise in the raw data. Kalman filtering is an efficient optimal estimator given the measurements observed over time. The Butterworth filter finds the best compromise between phase response and attenuation. The average filter calculates the average output sample from a finite number of input samples. We concluded that the average filter was sufficient for obtaining a smoothing signal in this work.

The system recorded 1400 data samples, of which 80% were used for the training dataset and 20% for the testing dataset. The performance of the models was evaluated through confusion matrices. We randomly selected 280 breathing samples (20%), consisting of 80 normal breath samples, 70 deep breath samples, 70 rapid breath samples, 40 cough samples, and 20 breath-holding samples as a test dataset to evaluate the model. Figure 6d shows that the recognition accuracy was up to 94% for the training and 92% for the testing datasets. In these matrices, NB represents normal breathing, DB represents deep breathing, RB represents rapid breathing, CO represents cough, and BH represents breath-holding. The cough and breath-holding types were the easiest to distinguish, while the rapid and deep breath types were easily confused with the normal type. Among them, the accuracy of the breath-holding type was the highest at 100%. The accuracy of the rapid breath type was the lowest at 87%. Through the above application, the sensor demonstrated potential for daily health-monitoring tasks.

## 5. Conclusions

In summary, we have proposed a respiratory monitoring system from a flexible resistive pressure sensor, an embedded module, and a random forest algorithm. The sensor was assembled from Pyralux film, CNT wipes, and thin adhesive layers. This thin structure demonstrated superior performance with a sensitivity of 0.2 kPa^−1^ (<6 kPa) and high water resistance. The potential of the sensor was evaluated in a practical system with various components. Among the challenges a system needs to address, the connections (between the flexible sensors and the electrical wires) and the signal processing model (which analyzes the resulting data) are two problems that need to be considered in detail. Here, the random forest algorithm was applied to recognize the different types of respiration, such as normal breath, deep breath, rapid breath, cough, and breath-holding. A model achieved a good accuracy of up to 92%, opening some new directions for studies in the future. The soft sensing system and machine-learning combination will become a universal platform for various wearable applications.

## Figures and Tables

**Table 1:** Comparison of some developed flexible pressure sensors.

Ref.	Principle	Thickness (µm)	Response Time (ms)	Sensitivity (kPa^−1^)	Water Resistance
[31]	Resistive	-	200	~7.12	Yes
[22]	Capacitive	~290	~41	0.23	Yes
[32]	Resistive	150	-	~0.001	No
[33]	Capacitive	~110	180/120	~0.14	No
[34]	Resistive	1000	-	~0.13	No
[35]	Capacitive	>1000	~100	0.18	No
[36]	Capacitive	>1000	~100	0.0124	No
Ours	Resistive	260	70/50	0.2	Yes

## Data Availability

The datasets generated and/or analyzed during the current study are available from the corresponding author upon reasonable request.

## Supplementary Materials

The following supporting information can be downloaded at: https://www.mdpi.com/article/10.3390/mi14091726/s1, Figure S1: Universal testing machine (UTM), consisting of a computer, a force load cell, an LCR meter, and a sole plate; Figure S2: Signal of the sensor when immersed in water for different durations (0, 6, or 12 h); Figure S3: SEM picture of Pyralux film after etching solution; Figure S4: Durability of the sensor (in the bending state with R = 22.5 mm) after 2000 cycles; Figure S5: The resistance change in the sensor at different temperatures.
