# Supplementary material for: Health Monitoring System from Pyralux Copper-Clad Laminate Film and Random Forest Algorithm
Source: Micromachines (Basel). 2023 Sep 1;14(9):1726. doi: 10.3390/mi14091726 (PMC10537244; doi:10.3390/mi14091726)
Supplement: Supplementary file 1 [file micromachines-14-01726-s001.zip › micromachines-2575486-supplementary.pdf]

micromachines

MDPI

*Supplementary Materials*

# Health monitoring system from pyralux copper-clad laminate film and random forest algorithm

**Chi Cuong Vu [1], Jooyong Kim [2], and Thanh-Hai Nguyen [1*]**

1 Faculty of Electrical and Electronics Engineering, Ho Chi Minh City University of Technology and Education, 01 Vo Van Ngan Street, Linh Chieu Ward, Ho Chi Minh City 700000, Viet Nam; cuongvc@hcmute.edu.vn, nthai@hcmute.edu.vn

2 Department of Materials Science and Engineering, Soongsil University, Seoul 156-743, Republic of Korea; jykim@ssu.ac.kr

* Correspondence: nthai@hcmute.edu.vn (Dr. Thanh-Hai Nguyen)

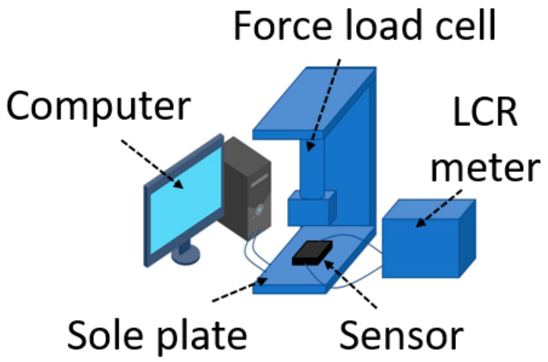


**Figure S1.** Universal testing machine (UTM), consisting of a computer, a force load cell, a LCR meter, and a sole plate.

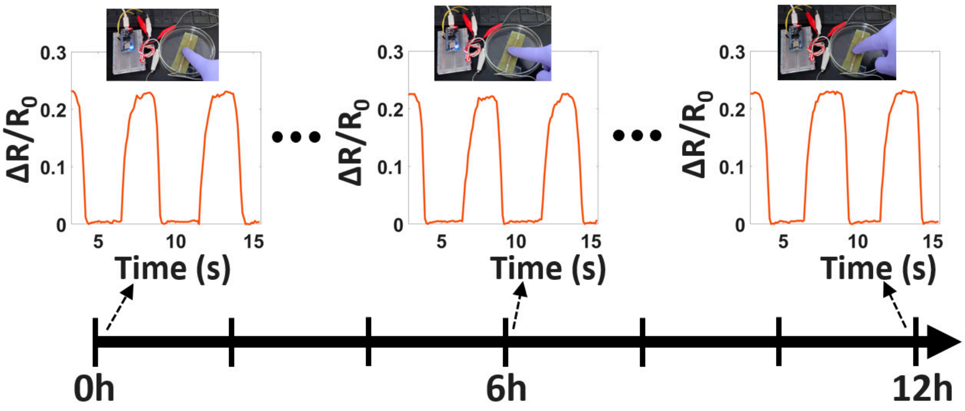


**Figure S2.** Signal of the sensor when immersed in the water at different times (0 - 6 - 12 hours).

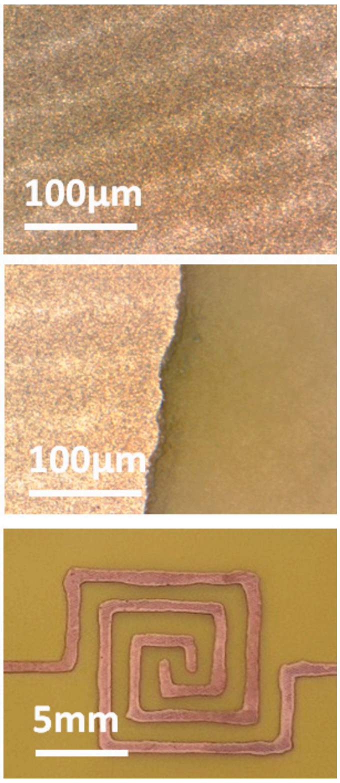


**Figure S3.** SEM picture of the pyralux film after etching-solution.

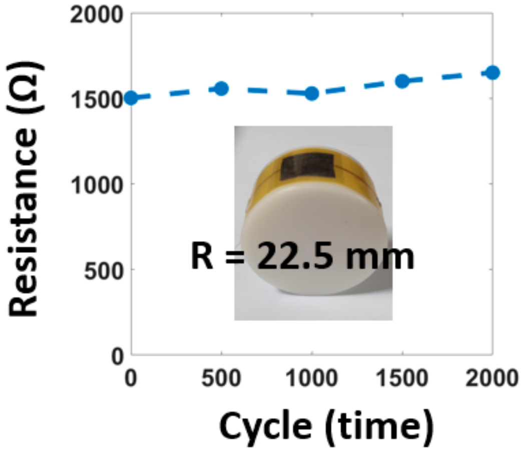


**Figure S4.** Durability of the sensor (in the bending state with R = 22.5 mm) after 2000 cycles.

The durability of the sensor (in the bending state with R = 22.5 mm ) is described in Figure S4. The maximum resistance was recorded every 500 cycles. The results show that the change is about 10 % after 2000 loading/unloading cycles.

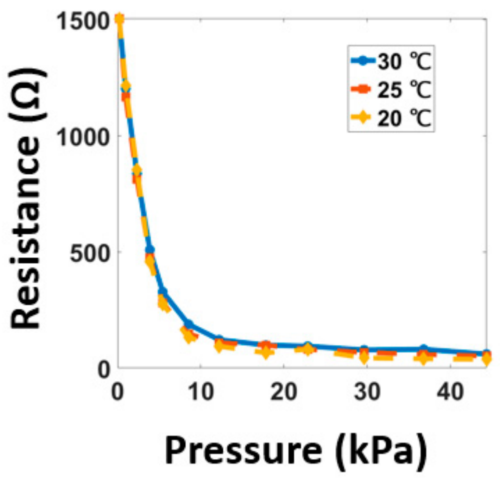


**Figure S5.** The resistance change of the sensor at different temperature.

Figure S5 shows the good stability of the sensor with different working temperatures (20 - 25 - 30 ℃). The water-resistant construction (polyimide layer and 3M adhesive layer) is to ensure that the sensor's operation is less affected by warmth (in breath) when monitoring human respiration.
